# Lipid oxidation in foods and its implications on proteins

**DOI:** 10.3389/fnut.2023.1192199

**Published:** 2023-06-15

**Authors:** Lianxin Geng, Kunlun Liu, Huiyan Zhang

**Affiliations:** ^1^College of Food Science and Engineering, Henan University of Technology, Zhengzhou, China; ^2^School of Food and Reserves Storage, Henan University of Technology, Zhengzhou, China; ^3^Zhengzhou Ruipu Biological Engineering Co., Ltd, Zhengzhou, China

**Keywords:** lipid, protein, oxidation, protein aggregation, free radicals

## Abstract

Lipids in foods are sensitive to various environmental conditions. Under light or high temperatures, free radicals could be formed due to lipid oxidation, leading to the formation of unstable food system. Proteins are sensitive to free radicals, which could cause protein oxidation and aggregation. Protein aggregation significantly affects protein physicochemical characteristics and biological functions, such as digestibility, foaming characteristics, and bioavailability, further reducing the edible and storage quality of food. This review provided an overview of lipid oxidation in foods; its implications on protein oxidation; and the assessment methods of lipid oxidation, protein oxidation, and protein aggregation. Protein functions before and after aggregation in foods were compared, and a discussion for future research on lipid or protein oxidation in foods was presented.

## 1. Introduction

**Figure 1:**
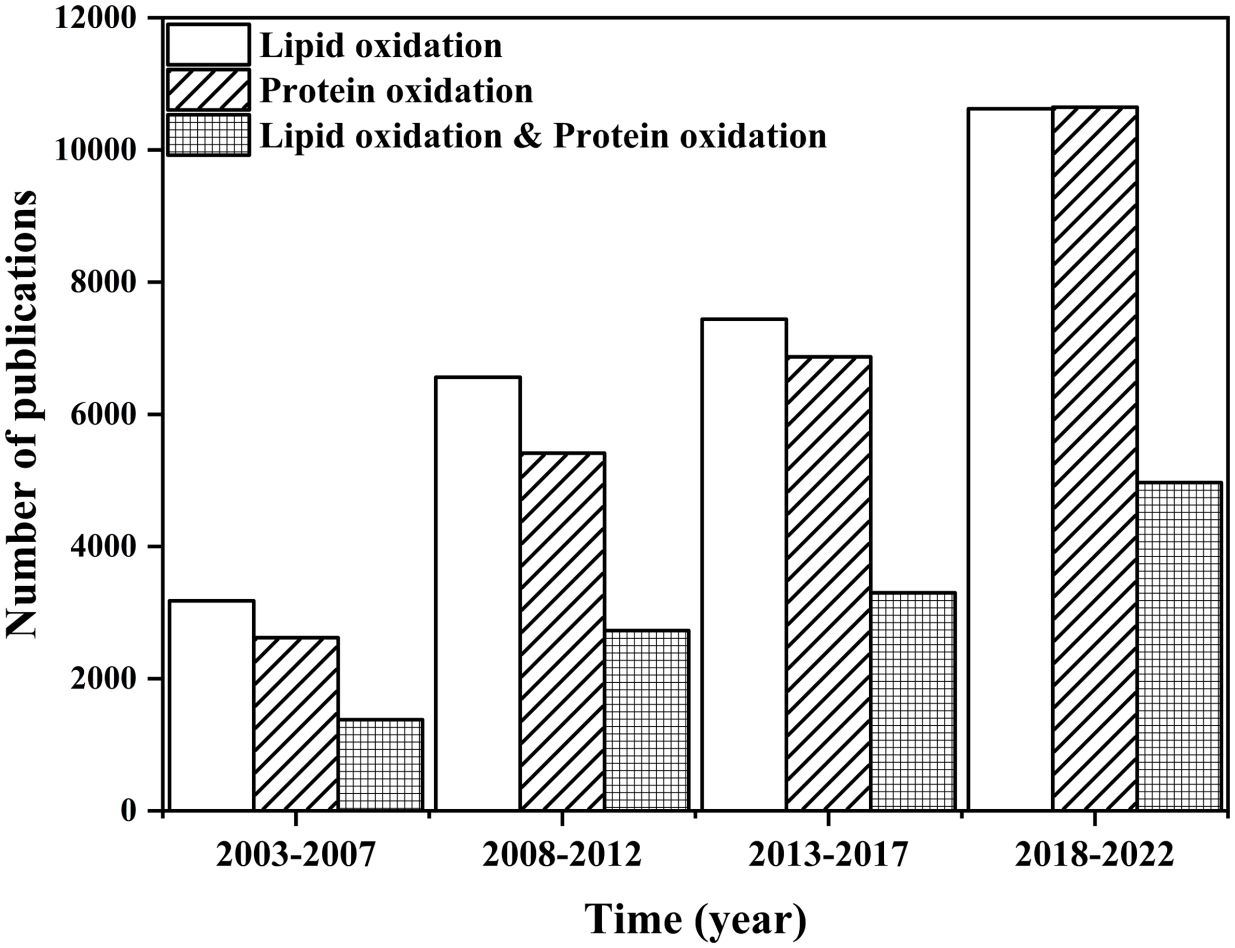
Comparison of the number of publications on “lipid oxidation,” “protein oxidation,” “lipid oxidation & protein oxidation” from 2003 to 2022. Data were summarized from the Web of Science.

Nowadays, food quality has attracted a considerable amount of attention. Food components and products undergo many chemical reactions during food processing, transportation, and storage. Since many nutrients are unstable, especially lipids and proteins, investigating their variation from food processing to storage is essential (1, 2). Since 2003, relevant research on lipid or protein oxidation has rapidly increased, especially in the last 3 years, revealing that researchers emphasized the variation of food nutrients (Figure 1). Lipid autoxidation, a continuous free-radical chain reaction, could cause an unstable and reactive food system, especially in meat (3). The free radicals in the food system could lead to protein oxidation, which could affect the protein structure by converting sulfhydryl to disulfide bonds (4). Lipid oxidation products could accelerate protein oxidation and subsequently induce protein aggregation (5). After excessive oxidation treatment, egg white protein aggregated, which was caused by a representative lipid oxidation product, 2,2′-azobis (2-amidinopropane) dihydrochloride (6).

However, research on the structural and functional change in proteins caused by lipid oxidation is limited (Figure 1). Oxidation could remarkably influence protein function (7). Protein oxidation not only affects the structure of the protein but also alters the physicochemical, techno-functional, and nutritional perspectives and even has critical implications on human health and safety (8–10). Therefore, it is essential to reveal the relationship between lipid oxidation and protein oxidation and its implication on proteins. To this end, the mechanism of lipid oxidation or protein oxidation was summarized, and the effect of oxidation in foods was discussed. The methods used to evaluate the impact of protein aggregation were also discussed.

## 2. Lipid oxidation in foods

### 2.1. Mechanisms of lipid oxidation

**Figure 2:**
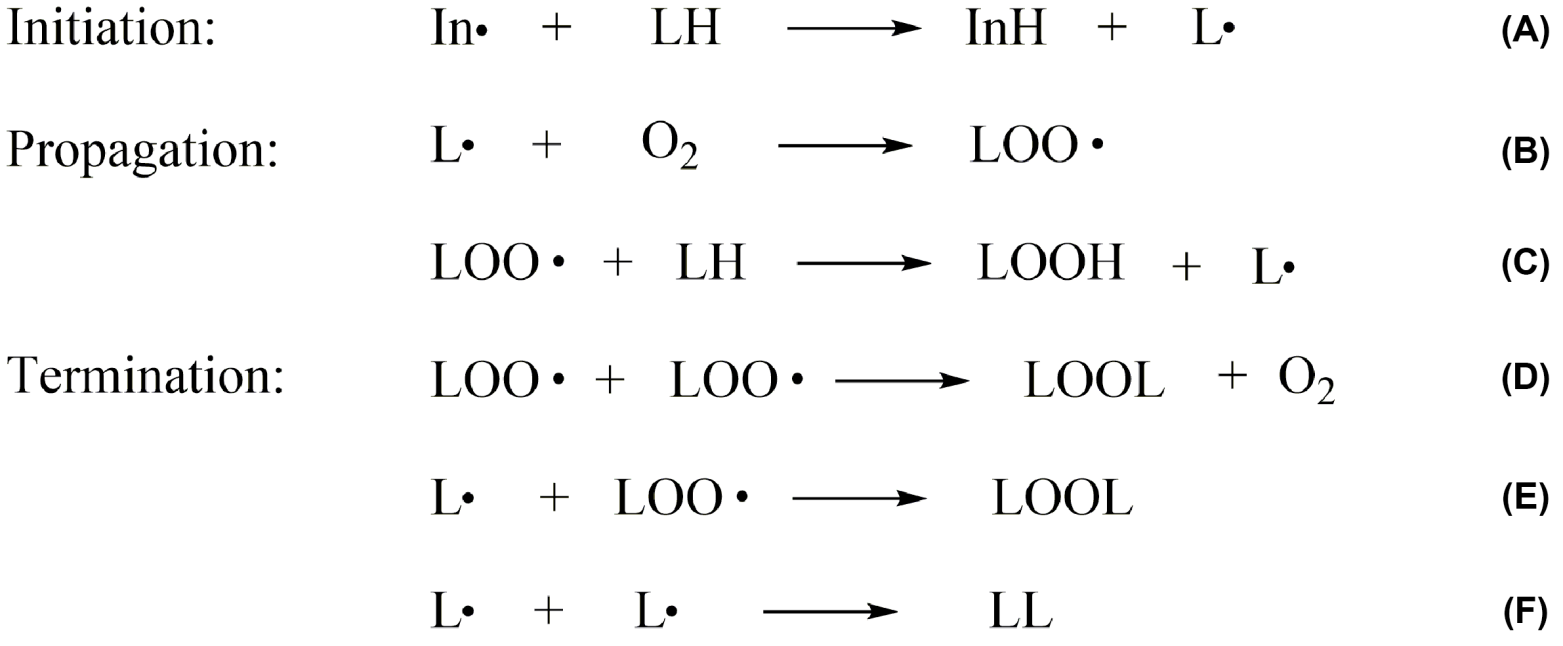
The mechanism of lipid auto-oxidation in food. **(A)** is the initiation period, **(B–C)** are the propagation period and **(D–F)** are the termination period of lipid oxidation. In∙: the free radicals; LH: the unsaturated fatty acid molecules; LOO∙: the lipid peroxyl radical; L∙: the lipid free radicals; O_2_: oxygen; LOOH: the lipid hydroperoxides; LOOL, LL: the lipid polymers.

Lipid oxidation is one of the leading causes of food spoilage. It refers to how unsaturated fatty acids in fats are slowly oxidized when exposed to oxygen in air, light, and metal ion. It includes auto-oxidation, photooxidation, and enzymatic oxidation (11). Auto-oxidation is a free-radical chain reaction, the primary interaction between unsaturated fatty acids and oxygen (12). In the initiation period, oil molecules produce free radicals under the effect of light, heat, or metal catalysts (Figure 2A). The propagation period and termination period of the free-radical chain reaction are followed (Figures 2B,C). The products of free radical and non-free radical reaction compounds are still free radicals. Only the non-free radical compounds are formed when free radicals react with free radicals, and the chain reaction is terminated (Figures 2D–F). Rancidity is triggered when lipid auto-oxidation accumulates to a certain degree (13). It could also produce aroma substances formed by large amounts of carbonyls, which contribute to the formation of meat characteristics and flavor (14).

**Figure 3:**
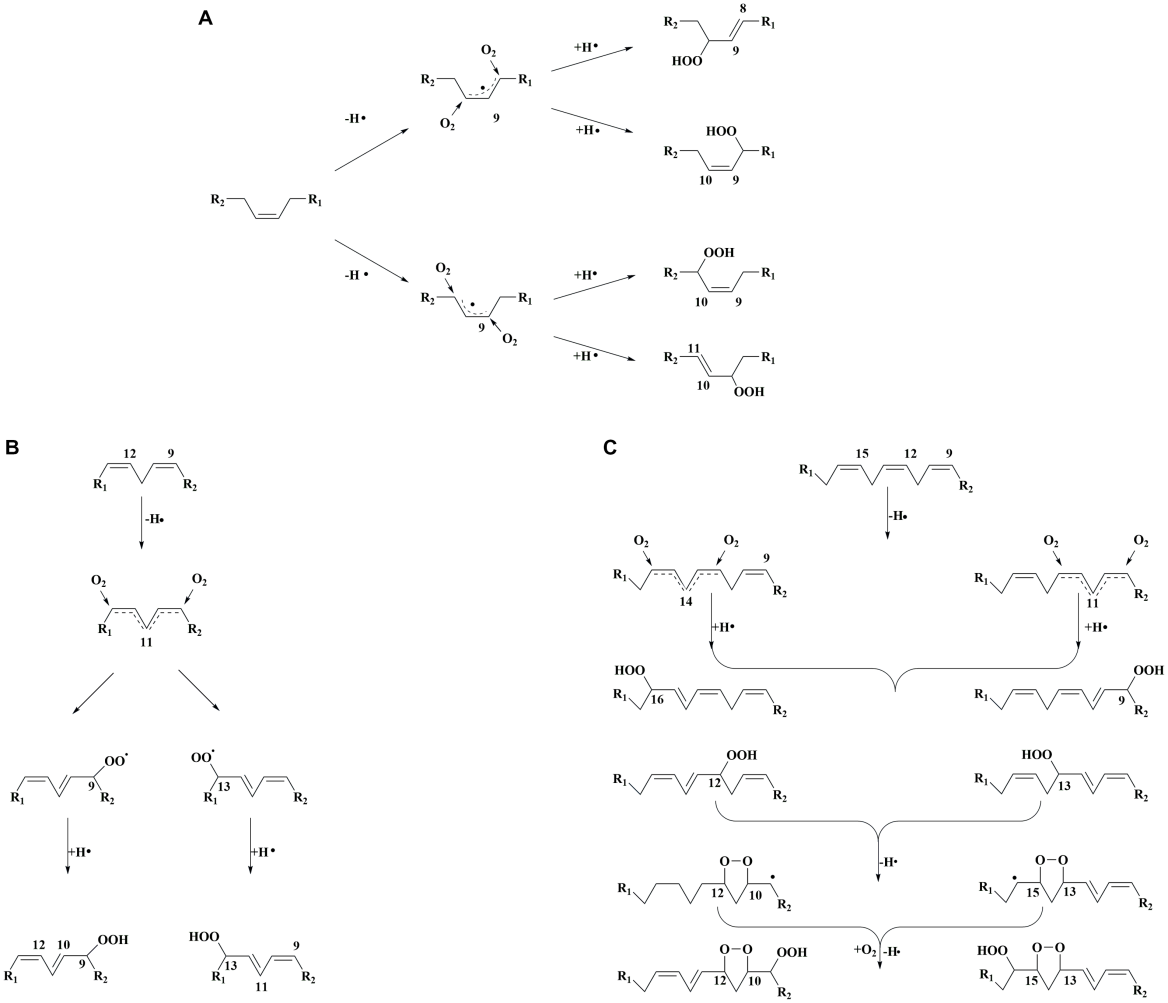
**(A)** Classical mechanism for oleic acid autooxidation. **(B)** Classical mechanism for linoleic acid autooxidation. **(C)** Classical mechanism for α-Linolenic acid autooxidation.

Hydroperoxides are the main products of lipid auto-oxidation, and the oxidation of different fatty acids could produce several hydroperoxides (15). The hydroperoxide was formed by free radicals, including the removal of a hydrogen atom from the α-methylene group of the double bond in the lipid. In this process, allyl radicals would be further formed. The electrons on allyl radicals could be delocalized at three carbon atoms, as in oleic acid, or delocalized at five carbon atoms, as in linoleic acid. For oleic acid (Figure 3A), the hydrogen leaving on C8 and C11 penta-dienyl could generate two allylic radicals. Moreover, 8-, 9-, 10-, and 11-allyl hydroperoxides could be caused by the reaction of intermediates with oxygen (16). The linoleic acid auto-oxidation involves doubly reactive, in which penta-dienyl radicals could be formed by the allyl groups of C11 (16) (Figure 3B). The conjugated 9- and 13-diene hydroperoxides could be formed by the reaction of intermediates and oxygen. Linolenic acid could form two penta-dienyl radicals by abstracting hydrogen on the C11 and C14 methylene groups (16) (Figure 3C). In addition, unsaturated fatty acids are active with singlet oxygen, which could increase the number of double bonds and make the food system unstable (17).

**Figure 4:**
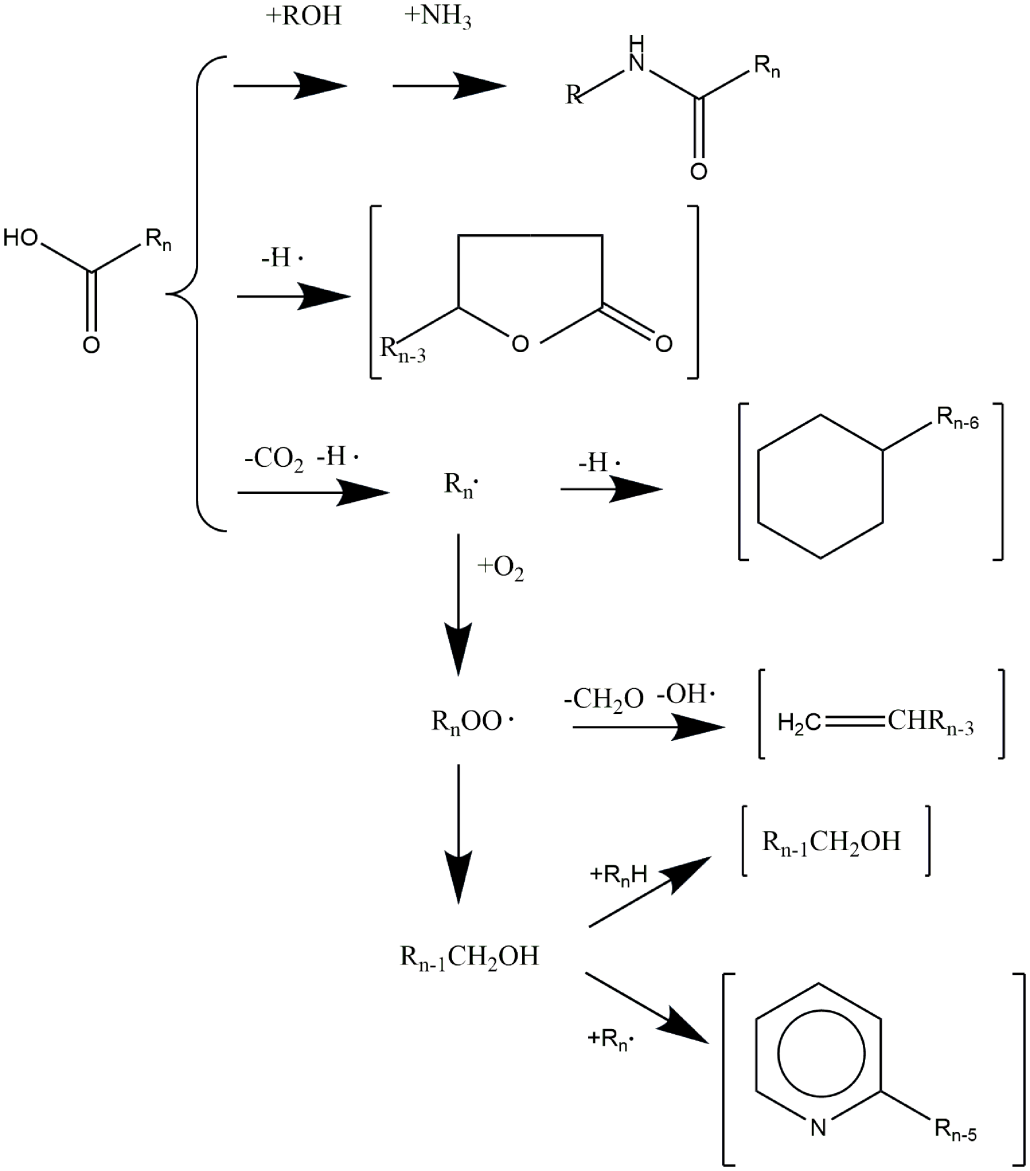
The mechanism of aroma compounds formed during lipid oxidation.

The degradation products of hydroperoxides depend on temperature, pressure, and oxygen concentration. Hydroperoxide cleavage could generate various volatile aromas and non-volatile substances (18). Some degradation products always affect the aroma and odor of cooked or stored meat products (19). Hydroperoxide degradation could generate alkoxy and hydroxyl radicals due to the homogeneous cleavage of OOH. The alkoxy radicals are cleaved on the C-C bond to form aldehydes and vinyl radicals or unsaturated aldehydes and alkyl radicals and then form volatile organic compounds such as aldehydes, alkenes, and alcohols (Figure 4). Among substances generated by the cleavage of alkoxy radicals, aldehydes are the essential critical aroma substances (20). The products formed by the cleavage reaction depend on the stability of the fatty acids in foods and the degradation products of hydroperoxide isomers (21).

### 2.2. Effect of lipid oxidation in foods

Lipid oxidation is vital to food quality during food processing and storage. The oxidation of lipids, especially in poly-unsaturated fatty acids, entails the generation of rancid or off-flavor, decreases the nutritional value, and reduces the storage period of foods (14, 22). The oxidation reactions of proteins and lipids could lower rice’s cooking, nutritional quality, and commodity value, including the loss of flavor, color, or nutrient value and functionality (23). Oxidation products (including primary, secondary, and tertiary oxidation products) accumulating to a certain extent could be detrimental to consumer health. In meat, the products formed by lipid oxidation, such as H_2_O_2_, peroxynitrite, hydroxyl radicals, and reactive aldehyde groups, play a role in myosin damage, further affecting the skeletal muscle components and lead to changes in the physical and functional properties of myosin (24). According to Zhou et al. (25) long-lived myofibrillar protein radicals were formed by oxymyoglobin oxidation caused partly by lipid oxidation. In addition, aldehydes, as the products of lipid oxidation, are closely related to the deterioration of meat color and flavor and muscle loss (26). Lipid oxidation occurs not only in animal-based foods but also in plant-based foods, thus it should not be ignored. Lipid oxidation could decrease rice breakdown, decreasing starch viscosity during storage (23). In addition, glutelin and lipid oxidation could affect rice quality, such as whiteness and aroma (27).

### 2.3. Assessment methods of lipid oxidation in foods

**Figure 5:**
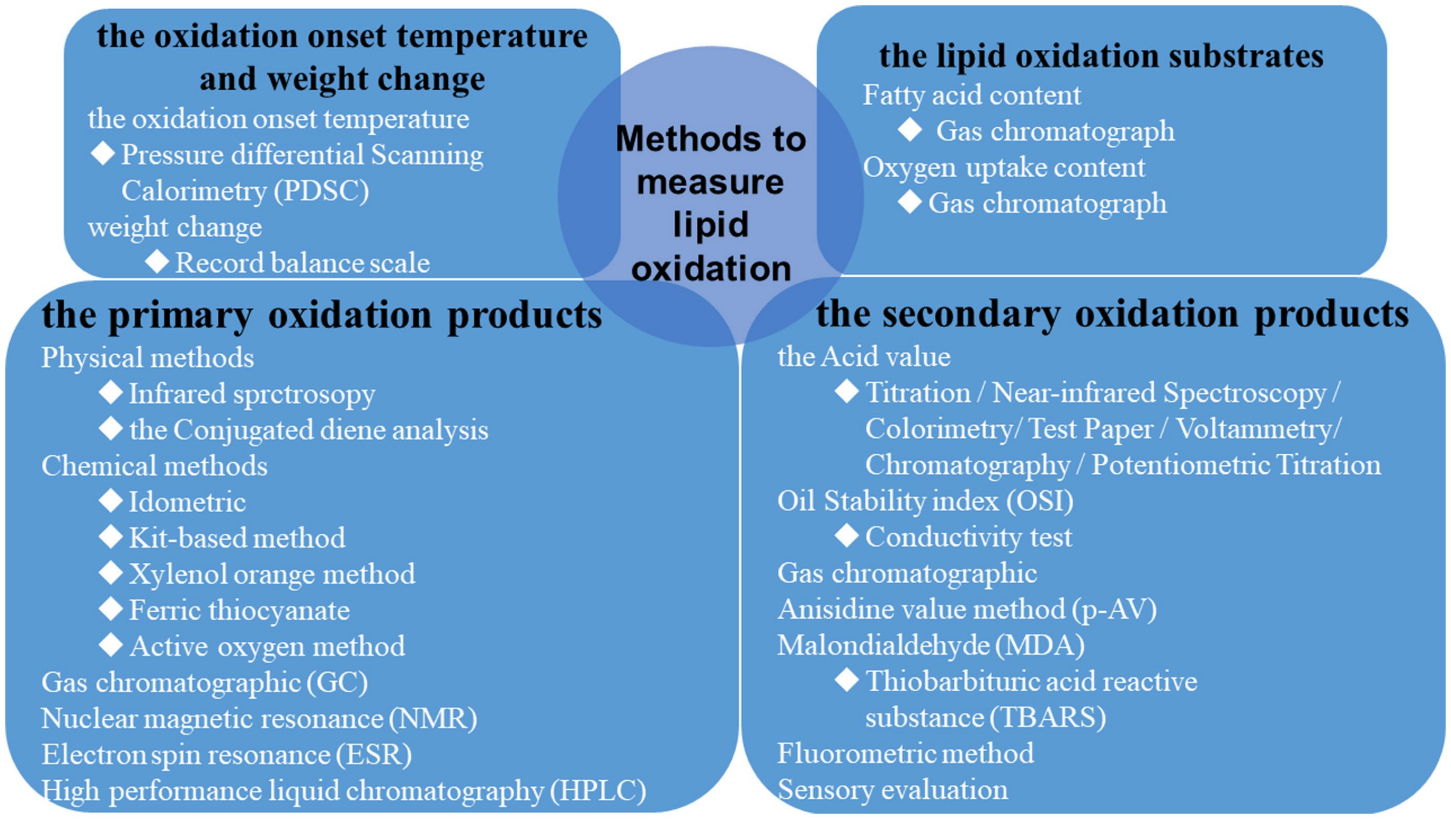
Summary of standard methods used to measure the degree of lipid oxidation.

Quantitative determination of the degree of lipid oxidation could provide the essential technical basis for evaluating food quality. The existing techniques correspond to the measurement of oxidation products and the consequences of lipid oxidation products (Figure 5) (28). Choosing an appropriate measurement to study the degree of lipid oxidation in foods is necessary. The following methods are commonly selected for the primary oxidation products to analyze lipid oxidation. The physical methods include infrared spectroscopy and conjugated diene analysis, and the chemical processes include peroxide value (PV) measurement, xylenol orange method, and active oxygen method (29, 30). In addition, the primary oxidation products of lipid oxidation can also be detected by high-performance liquid chromatography, nuclear magnetic resonance (NMR), gas chromatography (GC), and electron spin resonance (31–33). In foods, the secondary products of lipid oxidation are commonly measured by detecting the acid value, oil stability index, and malondialdehyde (MDA) (29). Many methods are used to measure the acid value, including titration, test paper, colorimetry, chromatography, near-infrared spectroscopy, potentiometric titration, and voltammetry (34). The secondary products could also be detected by GC, fluorometric method, and sensory evaluation (19, 35). Besides, the lipid oxidation substrates, weight change, and the oxidation onset temperature are used (36). Several of these methods are presented in detail below.

PV is commonly used for primary oxidation products to determine the peroxide content in foods, especially meat (37). The iodometric and ferric thiocyanate methods determine the PV in foods, which could directly measure the degree of hydroperoxides formed by oxidation (38). The iodometric assay is highly sensitive and accurate, and it is also suitable for minimum apparatus. However, in this experimental method, the oxygen in the reaction solution must be minimized (39). Reducing the generation of substances that may induce hydroperoxides decomposition or react with iodine is necessary to take precautions when precisely analyzing the degree of lipid oxidation. For insect-based food, the ferric thiocyanate method is more straightforward than the iodometric method (40). However, this method is not suitable for long-term storage of meat, especially ground meat, because long-term storage could re-decompose the hydroperoxide produced in meat, which could then affect the accuracy of PV.

Moreover, conjugated diene analysis (28) measured at 233 nm is suitable for polyunsaturated fatty acid-containing foods. It could provide the actual values of low-density lipoprotein oxidation during the early stage. It is convenient and low cost, but it depends on the lipoproteins’ composition and size. In addition, small conjugated dienes are challenging to detect. In methods of detecting the secondary oxidation products, the thiobarbituric acid reactive substances assay is commonly used. The production of MDA and TBA could be detected at 532 nm (41, 42). This method detects meat and meat-based products, fish and fish-based products, and edible insects. In addition, chromatography and fluorometric methods are sensitive, fast, and accurate, but they are costly to widely use (43, 44). Sensory analysis could provide the overall quality of food, and it could be used for liquid, semi-solid, and solid foods (45). However, it is limited by the participants and the change of time. Furthermore, the primary and secondary oxidation products could be determined by the p-anisidine value test and total oxidation index methods (46). They are simple calculations to test oil and oil-based products, but they are troubled with detecting omega-3-rich oils that contain specific flavorings (47).

## 3. Protein oxidation in foods

The variation of food function and deterioration caused by protein oxidation has recently become research highlights. For meat products, protein oxidation could reduce sensory characteristics, such as tenderness, flavor, and color, and break the functional properties, such as gelatinous and emulsification (48). Xia et al. (49) studied the effect of H_2_O_2_ concentration in the hydroxyl radical oxidation system on the degree of protein oxidation and the characteristics of myofibrillar protein gel whiteness, water holding capacity, texture properties, and elastic modulus. With increasing H_2_O_2_ concentration, the carbonyl value of myofibrillar protein increased, and the protein oxidation intensified. Similarly, in the hydroxyl radical oxidation system of Peruvian squid, when the oxidation concentration increased, more severe damage could be found on the myofibril structure, and water retention decreased (24). Peptide bond cleavage, amino acid residue oxidation, and disulfide bond formation are typically caused by protein oxidation. Therefore, protein oxidation could be reflected by structural or molecular weight changes.

### 3.1. Mechanism of protein oxidation

**Figure 6:**
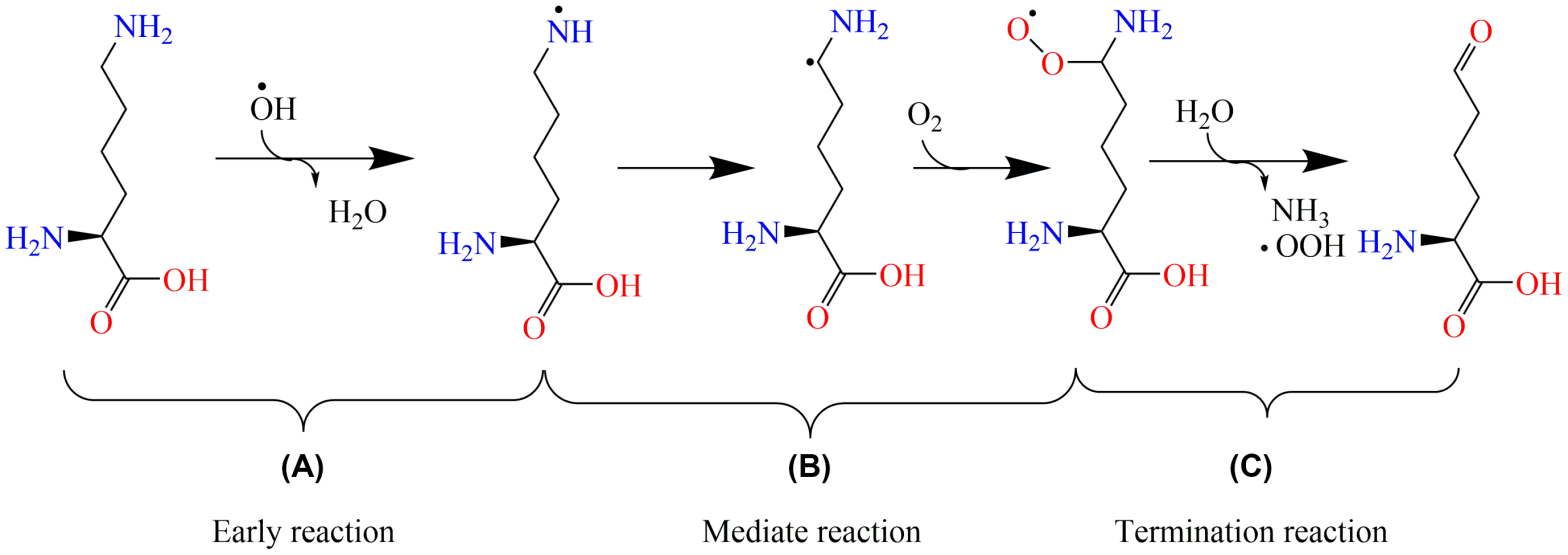
The oxidation mechanism of lysine. **(A)**, **(B)**, and **(C)** are the early, mediate, and termination reactions of protein oxidation, respectively. OH∙: hydroxyl radical; OOH∙: peroxyl radical.

Protein oxidation reactions are divided into radical beginning, intermediate, and termination reactions. During early reactions, protein radicals, and hydroperoxides are generated (Figure 6A). Intramolecular and intermolecular radicals are then transferred into peptides and proteins (Figure 6B). Non-radical products are formed during the termination reactions (Figure 6C). As the protein-specific structure formed by polypeptide chains is composed of dehydrated and condensed amino acids, the variation in oxidized protein is connected with amino acids, such as cysteine (Cys), methionine (Met), and lysine (Lys). Free radicals could be caused by the radical transfer reaction between amino acid residues, and they could lead to further oxidative damage in places that are not the initial position of oxidized proteins (50). For aliphatic amino acids, oxidation is generally carried out by abstracting hydrogen at the α-carbon atom to form a carbon-centered radical, such as arginine (Arg) (51). Aromatic amino acids, such as tryptophan (Trp) and tyrosine (Tyr), are easily oxidized (52). In addition, for amino acids such as Trp, Tyr, and Cys, the metal ion-catalyzed oxidation system could deteriorate the side chains of amino-acid residues (53). For example, the lipid oxidation-induced targets in Lys are lys-residue side chains (Figure 6B).

### 3.2. Assessment methods of protein oxidation in foods

In recent research, moderate protein oxidation could improve the functional properties of proteins. Moderately oxidized rice bran protein significantly changed the gut microbiota’s composition and improved the intestine’s barrier function (54). Moderate oxidation could also improve myofibrillar protein’s gelling capacities (49). However, highly oxidized protein shows an adverse phenomenon that could affect the structural characteristics, function, nutritional value, and even the body’s health (55). The degree of protein oxidation, which is caused by lipid oxidation, is often assessed by detecting the markers of protein oxidation. Changes in amino-acid levels induced by lipid oxidation products are often studied because of their high susceptibility. The oxidative modification of amino acids could reduce their bioavailability and nutritional value (56) (Table 1).

**Table 1:** Oxidation products of some primary amino acid monomers.

Amino acid	Original structure	Oxidative products	Condition	References
Cysteine (Cys)	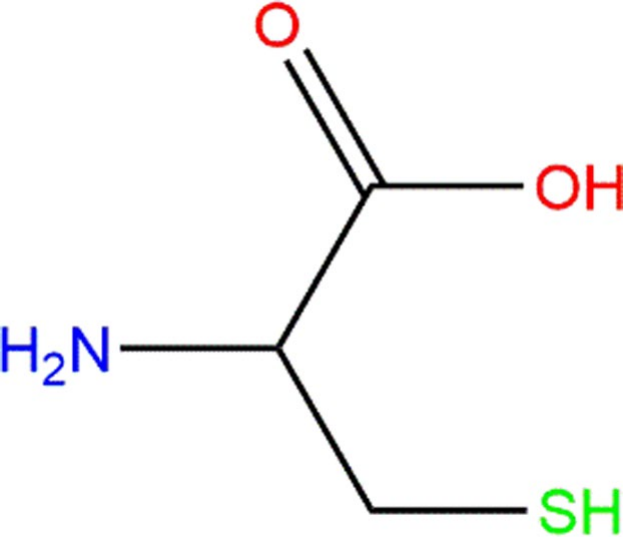	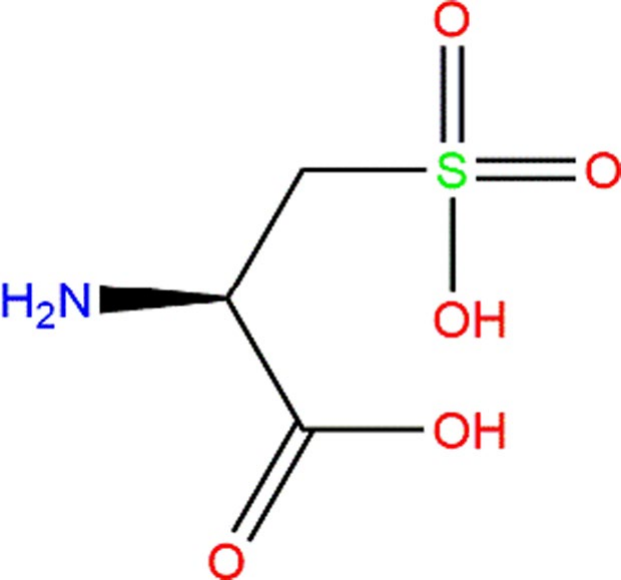	Heteroatoms are direct targets of radical formation.	(4, 57)
Tryptophan (Trp)	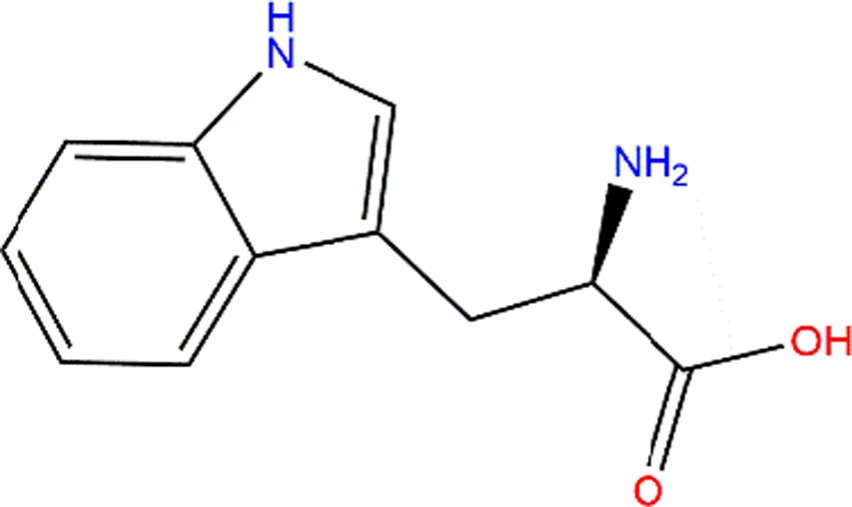	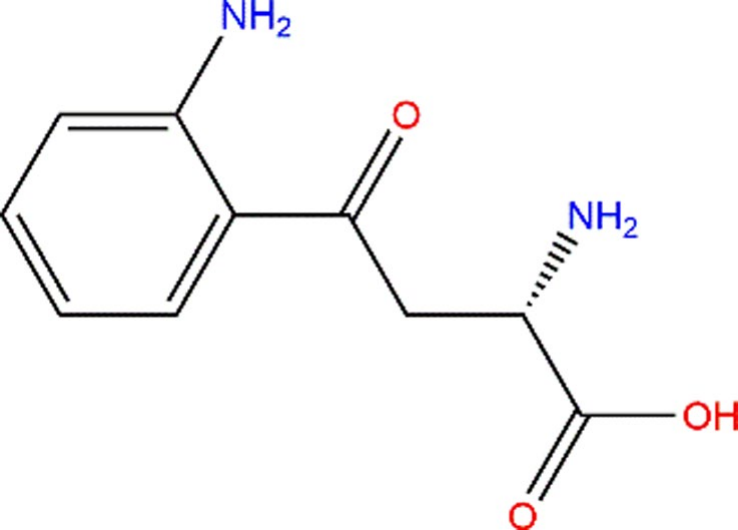	Exposed to UV or γ-irradiation.	(58, 59)
Methionine (Met)	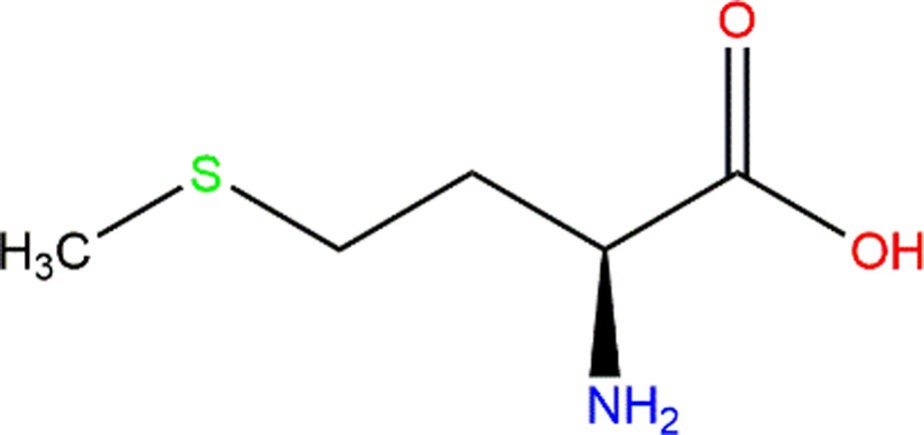	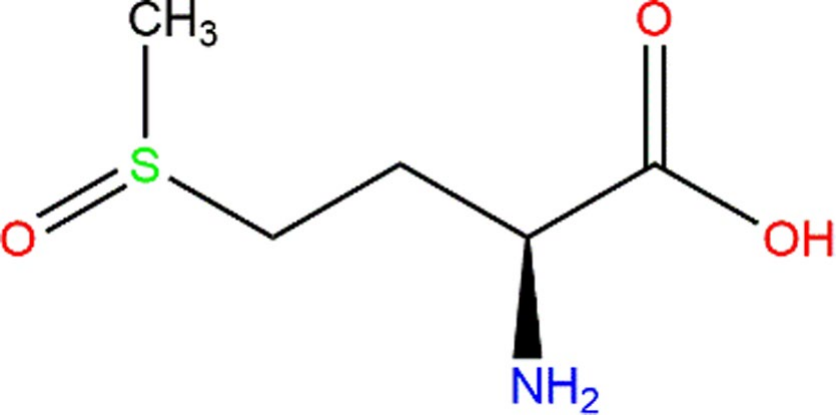	Heteroatoms are direct targets of radical formation.	(60)
Lysine (Lys)	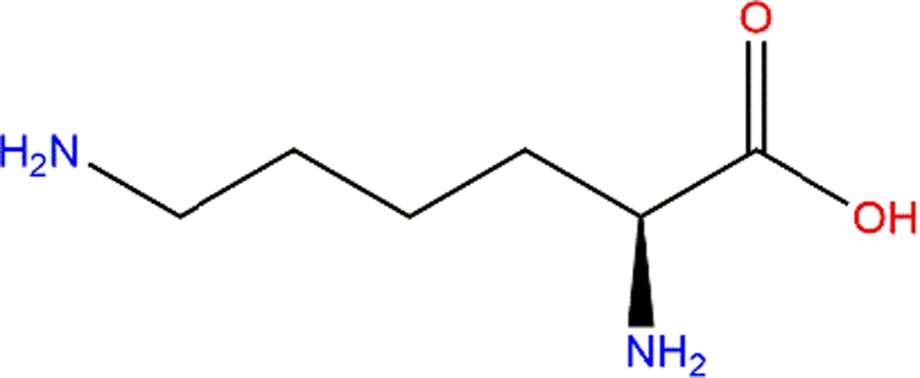	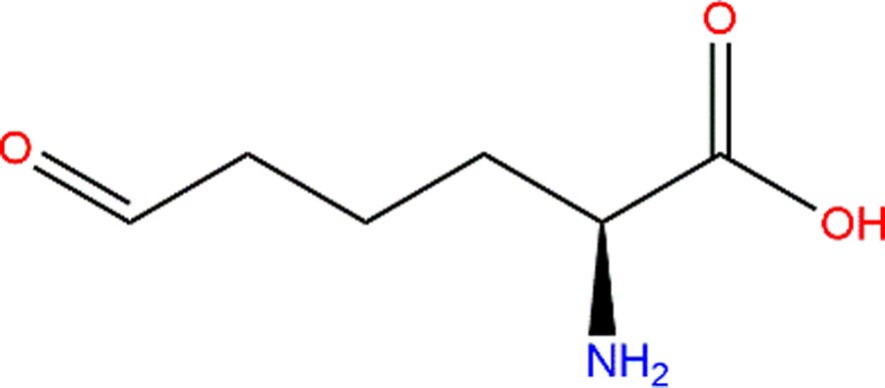	Fe^3+^ → Fe^2+^.	(61)

Cys is commonly used marker of protein oxidation in foods. Although the carbonyl content could not fully express the degree of protein oxidation, it could be further reflected by measuring the level of Cys. Under high temperatures, the free sulfhydryl content in fresh rice was higher than that of stored rice, and the free sulfhydryl content of rice decreased with protein oxidation (57). Similarly, when free radicals oxidize proteins, Tyr is sensitive to evaluating the oxidation degree. Under the action of free radicals, Tyr is oxidated to form di-tyrosine (58). Trp residues are also sensitive to oxidation and could generate an indolyl radical, which could react with Tyr or Cys residue (5). The fluorescence spectroscopy technique has always been used to detect the variation in Trp content. However, this method could not be used alone.

In recent research, protein oxidation could destroy protein secondary structure. The carbonyl group was found to be increased, and the sulfhydryl groups were lost in rancid rice bran (62). Otherwise, protein oxidation could be accelerated by lipid oxidation. Under the promotion of lipid oxidation, proteins’ surface hydrophobicity and rheological properties could change accordingly (63). Li et al. (64) found that with the increase in protein oxidation, the content of the stable secondary structure of α-helix and β-fold decreased. A trend that α-helix and β-fold could transfer to β-turn and the random coil was found, indicating that the structure of proteins has been destroyed. Surface hydrophobicity is one of the most critical factors in sustaining protein tertiary structure, and it is necessary to stabilize the protein structure and function (65). The molecular structure of oxidized protein could be folded, and the peptide tendon could break, thus enhancing the protein surface hydrophobicity. This phenomenon may be caused by inserting side chain groups of hydrophobic aliphatic and aromatic amino acids (66). Besides, the proteins were proven to be aggregated by protein oxidation (4). Protein aggregation may be a complete result of the formation of covalent cross-links, disulfide bonds, hydrogen bonds, and salt bridges. Especially under non-covalent interactions such as hydrophobic interaction, larger aggregates of proteins are formed (67).

## 4. Implications of lipid oxidation on proteins in foods

Free radicals and other small molecules, such as ketone and aldehyde, could be formed by lipid oxidation. The free radical reactions and carbonylation may be the mechanism that leads to the covalent binding of lipid peroxide products to proteins and lipid-induced protein aggregation (55). Proteins have been proven to be sensitive to free radicals. Protein oxidation could be induced by interacting with reactive oxygen species (ROS) or the by-products of oxidative stress (68). Free radicals formed by rancid rice bran could attack the main chain and side chain of proteins, and then protein oxygen-free radicals could be formed (69). The protein oxygen-free radicals induce a chain reaction with radicals or proteins. Subsequent oxidation of protein radicals could generate protein carbonylation caused by C-terminal decarboxylation and fragmentation in the skeleton. Crosslinking of myosin and light meromyosin was found in the hydroxyl radical generation system (55). Based on current research, protein carbonylation is mainly caused by the existence of amino-acid side chains (49). The side chains are susceptible to ROS, especially Lys, threonine, Arg, and proline. Besides, di-tyrosine is another possible crosslinking agent that may lead to protein aggregation in meat (70).

The rod sub-fragment of myosin is attacked by ROS first (71). The by-products of lipid oxidation, such as MDA, 4-hydroxy-2-nonenal (4-HNE), and acrolein (ACE), are electrophilic reagents that could react with nucleophilic groups in proteins. MDA could promote protein carbonylation and the loss of Trp fluorescence. Furthermore, during the oxidation of myoglobin and myofibrillar proteins, MDA could increase high-valent myoglobin species and reduce nonheme iron to affect ROS (26).

A series of reactions are induced by oxidative protein modifications (70), including biochemical changes and crosslinking formation. The biochemical changes include the variation of carbonyl compounds (72), emulsifying activity (67), and surface hydrophobicity (73). The crosslinking changes include the variation of di-tyrosine and disulfide bonds (74, 75). Protein oxidation could induce protein degradation and crosslinking (76, 77). It could also trigger various changes, such as modification of amino-acid side chains, peptide scission, structural unfolding, and protein depolymerization (78). Oxidized rice bran protein could accumulate oxidized products and decrease antioxidant enzymes, finally causing kidney injury in mice (79). In foods, because structure determines properties, the studies mainly focused on the reaction that oxidized proteins affect the functional properties of proteins (69) but overlooked the reaction mechanism of protein aggregation caused by oxidation. Therefore, the oxidative aggregation of proteins and their functional changes were described in detail.

## 5. Protein aggregation caused by lipid oxidation in foods

### 5.1. Effect of aggregated protein in foods

Protein oxidation could influence protein structure and change the protein’s original function. Table 2 summarizes the typical variation of protein function partially caused by oxidation. Solubility could become poor because the aggregated protein could form a compact spherical structure with accumulated disulfide bonds (84). For rice bran proteins, the increasing range of disulfide bonds and β-sheet could decrease the solubility, indicating that the proteins were directed to form insoluble aggregates (62). In addition, protein oxidation could simultaneously expose hydrophobic groups and facilitate protein crosslinking by hydrophobic interaction (85). The hydrophobic surface interactions were decreased, and the digestibility of pepsin and trypsin in rancid rice bran was lost (81). With the increasing protein oxidation, the foaming and emulsifying capacity decreased because of protein aggregation in rice bran (62).

**Table 2:** Variation of protein function caused by oxidation.

Function		Variation	Food source	Mechanism	References
Solubility		Loss of the water-holding capacity	Meat, rice bran	Intermolecular disulfide crosslinks	(5, 80)
Digestibility		Loss of pepsin and trypsin digestibility	Rice bran	Protein aggregation and crosslinks promoted through non-disulfide covalent bonds	(81)
Foaming		Slightly improve	Egg white protein	Modify molecular arrangement and interaction	(62, 82)
Color		Loss of redness	Muscle food (brown pigment)	Hemoglobin autoxidized to metHb	(1)
Texture	Gelling	Impaired	Meat	Protein carbonylation induced by Cu^2+^/systems	(71)
	Viscosity and elasticity	Tenderness decreased, but hardness, springiness, and gumminess improved under moderate oxidation	Meat	Protein thiols oxidized, excessive carbonylation of protein	(2, 5, 83)
	Cohesiveness	Improved to some extent	Meat	Depolymerization of myofibrillar proteins; unfolding of α-helix and exposure of the hydrophobic groups	(49)

In meat products, the color could be decreased due to the oxidation of myoglobin to metmyoglobin, indicating a decrease in the shelf-life of meat products (22). Besides color variation, gel hardness is negatively correlated with carbonyl group contents in oxidized protein gels, indicating the degradation of oxidized protein gels (85). Similarly, tenderness and water binding capacity decreased with protein oxidation in pork (86), and the oxidation of protein thiols could lead to protein aggregation and decreased tenderness (87). The effect of protein aggregation has been studied in current meat production, such as oxygen-modified atmosphere packaging (MAP). Under MAP in beef, the hardness of cooked parties (88) and the compression force of myofibrillar gel were found to be increased (89).

### 5.2. Assessment methods of aggregated protein in foods

**Figure 7:**
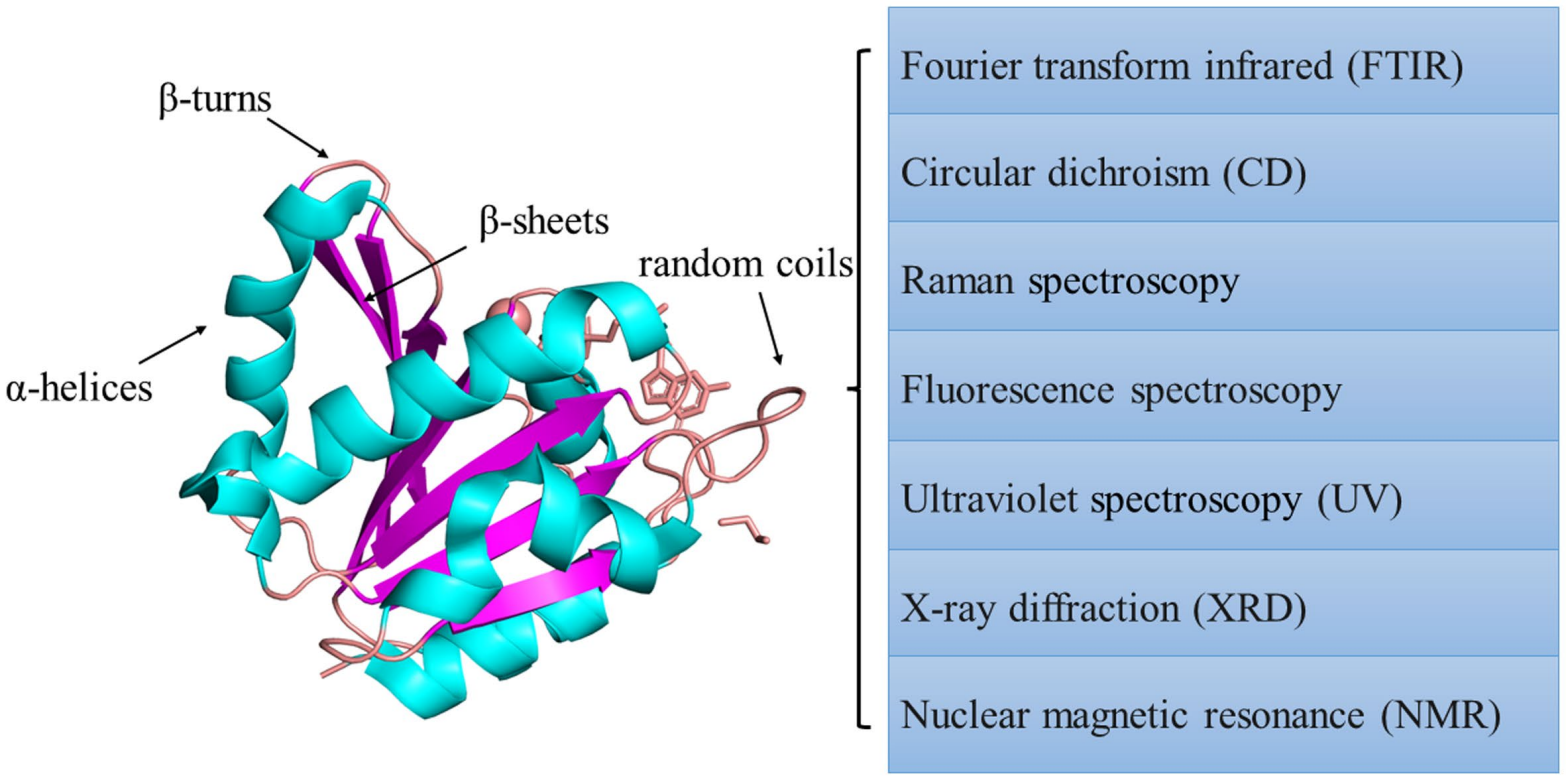
Summary of spectroscopic methods used to measure protein structure.

Protein aggregation could be caused by protein oxidation because of the protein’s unstable structure. The methods to evaluate oxidized protein variation have also been applied to assess the degree of protein aggregation. X-ray diffraction (XRD) and NMR methods are widely used to evaluate the tertiary structure. However, XRD requires high-quality protein single-crystal samples, and NMR is limited by molecular weight and the condition that the sample particles should be small enough. Scanning electron microscopy (SEM) (85) is another standard method for analyzing the structure of biological macromolecules. Although it could directly investigate the protein tertiary structure, the ways to evaluate the variation of protein secondary structure could also be meaningful. Considering aggregated protein can be formed by protein oxidation, oxidized proteins could be potentially used to evaluate protein aggregation. The biochemical changes, the surface hydrophobicity, and the crosslinking formation caused by oxidation could be reflected by di-tyrosine and disulfide bonds 4 (4, 70). As secondary protein bonds, disulfide bonds are related to the change in protein tertiary structure. So, the content of carbonyl and sulfhydryl could be analyzed to evaluate the oxidative extent of proteins. The carbonylation variation and the sulfhydryl content increased in rancid rice bran, indicating that the proteins aggregated caused by rice bran oxidation (62). The secondary structure formed by individual amino acids has been explicitly proven (90). The formation is caused by their side-chain function and environmental factors. The structure of α-helices is regarded as the default conformation. Due to steric clashes, the branch on the β-carbon atom always causes unstable α-helix, such as valine and threonine (91). β-strands, the relatively extended ordered structure, mainly contain steric residues. So, α-helices, β-sheets, β-turns, and random coils are often detected to evaluate the variation of protein structure. Many methods are available to detect these metrics. The interpretation of the protein secondary structure could be reflected by protein change in the infrared spectral region. The secondary structure of protein and polypeptide has nine characteristic absorption bands in the infrared spectral region. Among them, the key absorption band to study protein secondary structure is the amide I band, which is located in the range of 1600–1700 cm^−1^. The characteristic bands of α-helices, β-sheets, β-turns, and random coils are in the range of 1650–1658 cm^−1^, 1600–1640 cm^−1^, 1660–1695 cm^−1^, 1640–1650 cm^−1^, respectively (92). The content of the β-sheet could be analyzed by Fourier transform infrared method (62). The contents of amino-acid side chains and random coils were found to be increased by this method, indicating that aggregated proteins were formed (93). Under high storage temperature conditions (70°C), the rice protein secondary structure was destroyed with the order structure, such as α-helices and β-sheets, decreasing and the random coils increasing because of lipid and protein oxidation (4). However, the study pointed out that the amide I region only could not sufficiently provide information for structure quantitation, and it could be combined with the amide III region to study protein secondary structure more comprehensively (94). In addition to IR spectroscopy, many methods could be applied to study protein secondary structure (95), such as circular dichroism (CD), Raman (96), and ultraviolet (UV) spectroscopies (97) (Figure 7). CD and IR methods are commonly used to analyze polypeptide polymers, and the CD method is usually applied in aqueous solutions. However, any spectroscopic technique should not be used alone to guarantee accuracy.

The variation of proteins’ rheological properties could also be used to evaluate the tertiary structure. With increased temperature, proteins could form a three-dimensional gel network system to create a tight spatial structure. Gel forming ability and gel elasticity could be judged by measuring the rheological properties of proteins. They are always expressed by the elastic or storage modulus (G′). The larger the G′, the stronger the elasticity of the protein to form a gel (64). The loss modulus(G′′) corresponds to G′, and it is used to represent the viscous properties of the protein. Tan α is the ratio between G′′ and G′, and it is a comprehensive evaluation index for the viscoelasticity of a sample, reflecting the energy loss of each oscillation process. Lower values indicate that the gel network structure improved. According to texture profile analysis, the hardness, springiness, gumminess, and cohesiveness of isolated myofibrillar protein improved under moderate oxidation of protein (49). Moreover, Raman spectroscopy and NMR are also used to study aggregated protein’s structure and physicochemical properties (Table 3).

**Table 3:** Common spectroscopic methods to evaluate protein structure.

Target	Method	Common advantage	Applicable condition	References
Secondary structure	Fourier transform infrared (FTIR)	Rapid, convenient, and increased popularity	Based on C=O	(98)
Secondary and tertiary structure	Circular dichroism (CD)		Optically active sample, and applied in aqueous solutions	(95, 99)
	Raman spectroscopy		Based on -CO-NH-	(96, 99)
Tertiary structure	Fluorescence spectroscopy	Rapid, convenient, and increased popularity	Based on the absorption of UV or visible light of chromophores that can emit photons, like natural chromophores: Trp, Tyr and phenylalanine (Phe) residues	(95, 100)
	Ultraviolet spectroscopy (UV)		Based on aromatic amino acid residues	(97, 100)
	X-ray diffraction (XRD)		Does not allow for real time conformational transition	(101)
	Nuclear magnetic resonance (NMR)	High resolution	Suitable for low molecular weight proteins	(102, 103)

Protein molecular weight distribution variation is also used to measure protein oxidation. By liquid chromatography -20A liquid chromatogram method, the aggregation of rice bran protein caused by rancid rice bran was proven (69). In addition, the molecular weight of barley grains protein could be measured by matrix-assisted laser desorption ionization-time of flight mass spectrometry (104). Sodium dodecyl sulfate-polyacrylamide gel electrophoresis (26) is also a standard method to evaluate the variation of molecular protein weight. This method could analyze protein molecular weight by the variation of protein bands, especially in highly oxidized proteins (71).

## 6. Summary and outlook

Whether in plant- or animal-based foods, light or high temperature storage conditions always deteriorate nutrient substances, especially lipids, and proteins. During lipid oxidation, fatty acids will decompose into carbonyl compounds, unsaturated aldehydes, ketones and other substances, forming an unstable food system. During the lipid auto-oxidation process, free radical chain reactions can cause protein oxidation and deterioration, even leading to protein aggregation. The protein aggregation caused by lipid and protein oxidation are due to various factors. At present, the effects on lipid or protein oxidation have been sufficiently evidenced, and there are multiple detection methods to measure the degree of food oxidation. Therefore, further studies should focus on the variation of lipids and proteins simultaneously and study their interaction mechanism. Further research on novel formulation strategies to minimize lipid and protein oxidation should be studied, especially in plant-based foods. The mechanism of protein denaturation, the specific degree of denaturation caused by lipid oxidation degree, and the categories of oxidized products should also be studied in depth.

## Author contributions

LG: investigation, resources, methodology, and writing – original draft. KL: funding acquisition, methodology, supervision, and writing – review and editing. HZ: resources and methodology. All authors have read and agreed to the published version of the manuscript.

## Funding

This study was supported by the National Natural Science Foundation of China (32172259), the Natural Science Foundation of Henan Province (212300410033), the Program for the Top Young Talents of Henan Associate for Science and Technology (2021), and the Innovative Funds Plan of Henan University of Technology (2021ZKCJ03).

## Conflict of interest

HZ was employed by Zhengzhou Ruipu Biological Engineering Co., Ltd.

The remaining authors declare that the research was conducted in the absence of any commercial or financial relationships that could be construed as a potential conflict of interest.

## Publisher’s note

All claims expressed in this article are solely those of the authors and do not necessarily represent those of their affiliated organizations, or those of the publisher, the editors and the reviewers. Any product that may be evaluated in this article, or claim that may be made by its manufacturer, is not guaranteed or endorsed by the publisher.
